# Transcriptomic Analysis of the Anterior Silk Gland in the Domestic Silkworm (*Bombyx mori*) – Insight into the Mechanism of Silk Formation and Spinning

**DOI:** 10.1371/journal.pone.0139424

**Published:** 2015-09-29

**Authors:** Huaipu Chang, Tingcai Cheng, Yuqian Wu, Wenbo Hu, Renwen Long, Chun Liu, Ping Zhao, Qingyou Xia

**Affiliations:** 1 State Key Laboratory of Silkworm Genome Biology, Southwest University, Beibei, Chongqing, China; 2 College of Biotechnology, Southwest University, Beibei, Chongqing, China; Institute of Plant Physiology and Ecology, CHINA

## Abstract

Silk proteins are synthesized in the middle and posterior silk glands of silkworms, then transit into the anterior of the silk gland, where the silk fibers are produced, stored and processed. The mechanism of formation and spinning of the silk fibers has not been fully elucidated, and transcriptome analyses specific to the anterior silk gland have not been reported. In the present study, we explored gene expression profiles in five regions of silk gland samples using the RNA-Seq method. As a result, there were 959,979,570 raw reads obtained, of which 583,068,172 reads were mapped to the silkworm genome. A total of 7419 genes were found to be expressed in terms of reads per kilobase of exon model per million mapped reads ≥ 5 in at least one sample. The gene numbers and expression levels of the expressed genes differed between these regions. The differentially expressed genes were analyzed, and 282 genes were detected as up-regulated in the anterior silk gland, compared with the other parts. Functions of these genes were addressed using the gene ontology and Kyoto Encyclopedia of Genes and Genomes databases, and seven key pathways were enriched. It suggested that the ion transportation, energy metabolism, protease inhibitors and cuticle proteins played essential roles in the process of silk formation and spinning in the anterior silk gland. In addition, 210 genes were found differently expressed between males and females, which should help to elucidate the mechanism of the quality difference in silk fibers from male and female silkworms.

## Introduction

Silkworm (*Bombyx mori*) is one of the most important economic insects. Silkworm larvae spin silk and provide the raw materials of silk fabrics. The silk gland is the only organ for producing, storing and processing silk fibers. According to function and morphology, the silk gland can be divided into three main parts: anterior, middle and posterior silk gland (ASG, MSG and PSG, respectively). The MSG is also composed of three parts: anterior, middle and posterior MSG (AMSG, MMSG and PMSG, respectively). Fibroins and sericins are synthesized in the PSG and MSG, respectively, then pass into the ASG, where the liquid silk proteins are processed, transported into the spinneret and finally secreted [1].

The fibroins and sericins are the main components of the silk fibers. Silk fibroins are a 2300 kDa molecular complex, composed of heavy (H-fibroin) and light (L-fibroin) peptides and a P25 protein, with a 6:6:1 molar ratio [2]. Fibroins are coated by sericins, which are encoded mainly by *Ser1*, *Ser2* and *Ser3* genes, and the molecular components of the silk sericins differ among the different producing stages [3]. All these proteins are spun into the cocoon after being transported through the ASG, where they are manufactured with an attractive stage. However, the mechanism by which liquid silk proteins are transformed into solid silk fibers has not been elucidated. It is widely accepted that the protein conformation transits from the random coil or helical conformation to a beta-sheet with liquid–solid conversion, and that ions, shearing and pH are involved in the process. Particle-induced X-ray emission, inductively coupled plasma mass spectroscopy and atomic absorption spectroscopy have shown that the metal element contents increased from the PSG to the ASG—with the exception of calcium (Ca), which decreased significantly in the ASG [4]. Raman spectroscopy of the secondary structure suggested that shearing can increase the orientation and beta-sheet structure, and improve the mechanical properties of silk fibers [5]. Moreover, pH is also an important element for conformational change of silk proteins [6]. These advances provide important clues to, but are insufficient to reveal the mechanism—additionally, the genes involved in the process are unknown. Combining the former biological knowledge, we inferred that all the necessary condition changes would not affect silk fibers’ features without the existence of a permeable gland wall and many functional proteins. A useful way to study this may be by screening the protein components and gene expression trends in the silk gland, especially the ASG.

Proteomic and transcriptomic methods were used to investigate proteins and genes’ expression in different parts or development periods of the silk gland. Two dimensional gel electrophoresis methods were used to characterize the protein distribution around the silk gland, and 95 proteins were identified and classified, and some differentially expressed proteins were found [7, 8]. A shotgun proteomic analysis, mainly on the ASG, led to the identification of 1132 proteins, of which 33 cuticle proteins were found expressed in the ASG, and other proteins were categorized into seven groups [9]. Expressed sequence tags (ESTs) and microarray techniques were used to search the differentially expressed genes (DEGs) in the silk glands [10, 11]. RNA-Seq technology was used to compare the transcriptomes of the Ras1CA-overexpressed and wild-type silkworm PSGs, and 2636 DEGs were found [12]. Transcriptomic sequencing technology and gel-free-based proteomic techniques were applied to silk production and showed that some pathways concerning energy metabolism were enhanced but silk production was low [13]. RNA-Seq showed 32 DEGs in the transcriptomes of MSG/PSG of two domestic and two wild silkworms [14].

However, transcriptome analysis specific to the ASG has not been reported, and the mechanism of silk spinning remains to be fully elucidated. In this study, the RNA-Seq method was employed on the ASG and other parts of the silk gland, to gain insight into the mechanism of silk formation and spinning. The transcriptomes of the ASG, AMSG, MMSG, PMSG and PSG in male and female silkworms were respectively sequenced. The genome-wide gene expression profiles in each part of the silk glands were constructed and analyzed. The genes different expressed between the ASG and the other parts were explored and 282 genes were detected as up-regulated in the ASG. The function of these genes were studied, and seven pathways were found to be enriched. Genes differentially expressed between males and females were also discovered. These results will provide important reference data for future research on silkworm, especially on the silk gland, and provide essential theoretical basis for the mechanism of the silk formation and spinning.

## Materials and Methods

### Sample Preparation

Silkworm larvae (*Dazao*) were raised on fresh mulberry leaves, under regular conditions of temperature of 25°C and relative humidity of 70%–80%, in the professional room in the State Key Laboratory of Silkworm Genome Biology. Silk glands were dissected from fifth-instar day 3 larvae and cut into five parts according to morphological characteristics: ASG, AMSG, MMSG, PMSG and PSG. The S-shaped connection parts were discarded. Ten samples were prepared from ASG, AMSG, MMSG, PMSG and PSG of male and female larvae. The samples were placed in liquid nitrogen and stored at –80°C until use.

### RNA Sequencing

Total RNA was extracted from each sample by TRIzol (Invitrogen, USA) and chloroform. RNA was dissolved by 20 mM NaAc/0.5% SDS solution and was measured by spectrophotometer and gel electrophoresis. RNA samples were stored in a refrigerator at –80°C for sequencing libraries. The TruSeq RNA Sample Prep Kit (Illumina, USA) was used to construct the cDNA library for sequencing, following the Illumina protocol. Each cDNA sample was clustered on cBot (Illumina, USA) and then sequenced on HiSeq2000 (Illumina, USA) following the Illumina protocol. Raw data presented in this publication were deposited in the NCBI Short Read Archive (http://www.ncbi.nlm.nih.gov/sra/) and are accessible through SRA accession number: PRJNA284192.

### Data Analysis

Data from the HiSeq 2000 were transmitted into a computer server. CLC Genomics Workbench v5.5 was used to analyze sequencing data following the manufacturer’s instructions [15, 16]. Initially, the raw data were imported into the CLC software, selecting the Illumina data item. The adapters were sheared when being imported. Then, the data were trimmed and the quality determined. The qualified data were retained for mapping to the silkworm genome from SilkDB [17], and the CLC experiment design option was used to compare data between different parts of the silk gland. The RPKM (Reads Per Kilobase of exon model per Million mapped reads) was selected as the criterion to judge whether the gene was expressed and its expression level [18]. A gene with RPKM ≥ 5 was considered as expressed. The threshold fold-change ≥ 2, P-value < 0.05 and FDR < 0.001 were the criteria to judge up-regulated genes. The DEGs were classified and analyzed by gene ontology (GO; http://bioinfo.cau.edu.cn/agriGO/analysis.php) [19] and Kyoto Encyclopedia of Genes and Genomes (KEGG; http://www.kegg.jp/) methods.

### RT-PCR

Total RNA was isolated from the ASG, AMSG, MMSG, PMSG and PSG, according to the same protocol as the sample preparation. Then, PrimeScript RT reagent kits (RR037A, Takara) were used to reverse transcribe the total RNA. All the primers were designed using Primer v5.0 software, and synthesized by the BGI company (Shenzhen, PR China) (S1 Table). *Ribosomal protein L3* (*RPL3*, BGIBMGA013567), a silkworm housekeeping gene, was selected as a control.

The RT-PCR mixture was performed in 10-μl volume containing 1 μl of 10 × PCR buffer, 0.8 μl of dNTPs, 0.1 μl of rTaq, 1 μl of primerF, 1 μl of primerR, 2 μl of template and 4.1 μl of ddH_2_O. The PCR reaction was performed in an ABI PCR System 9700 (Applied Biosystems) as follows: 94°C for 4 min, 25 cycles of 94°C for 10 s, 60°C for 15s, and 72°C for 30 s, and then 72°C for 7 min; and stored at 12°C or used immediately for detection.

## Results and Discussion

### Overview of RNA-Seq Data

A total of 959,979,570 raw reads were obtained from 10 libraries by HiSeq2000. After the low-quality reads were filtered, there were 939,306,844 reads preserved, with an average trimming ratio 97.85%, out of which 583,068,172 reads were mapped to the silkworm genome. The total length of the RNA-Seq data was about 57 Gb, corresponding to 132-fold coverage of the silkworm genome (Table 1). In the ASG from male and female silkworms, about 100- and 80-Mb raw reads were obtained, respectively, and about 58.8- and 48.5-Mb reads were mapped to the silkworm genome, or about 13.4- and 10.9-fold coverage of the silkworm genome, respectively. The length of mapped reads from each of the 10 samples exceeded 40 Mb.

**Table 1:** RNA-Seq data of different parts of the silk gland.

Samples	Number of raw reads	Number of reads after trimmed	Trimming Percentage	Number of mapped reads	Mapping Percentage	Total length of the reads (bp)
ASG-M	100291546	97785590	97.50	58768125	60.10	5759276250
ASG-F	80886436	79066029	97.75	48489226	61.33	4742246303
AMSG-M	110473784	108867389	98.55	76039206	69.85	7512673553
AMSG-F	83264248	80931498	97.20	58679019	72.50	5662525334
MMSG-M	113740416	112158063	98.61	64710041	57.70	6399823055
MMSG-F	82873894	79503450	95.93	50659838	63.72	4853212480
PMSG-M	99333848	97691250	98.35	54989331	56.29	5421948037
PMSG-F	82600788	81021651	98.09	49745844	61.40	4894991050
PSG-M	75997128	74108719	97.52	41816104	56.43	4106341413
PSG-F	130517482	128173205	98.20	79171438	61.77	7766718068
TOTAL	959979570	939306844	97.85	583068172	62.07	57119755543

ASG-M, ASG of male silkworms; ASG-F, ASG of female silkworms; AMSG-M, AMSG of male silkworms; AMSG-F, AMSG of female silkworms; MMSG-M, MMSG of male silkworms; MMSG-F, MMSG of female silkworms; PMSG-M, PMSG of male silkworms; PMSG-F, PMSG of female silkworms; PSG-M, PSG of male silkworms; PSG-F, PSG of female silkworms.

### Genome-wide Gene Expression Profile in the Silk Gland

**Fig 1:**
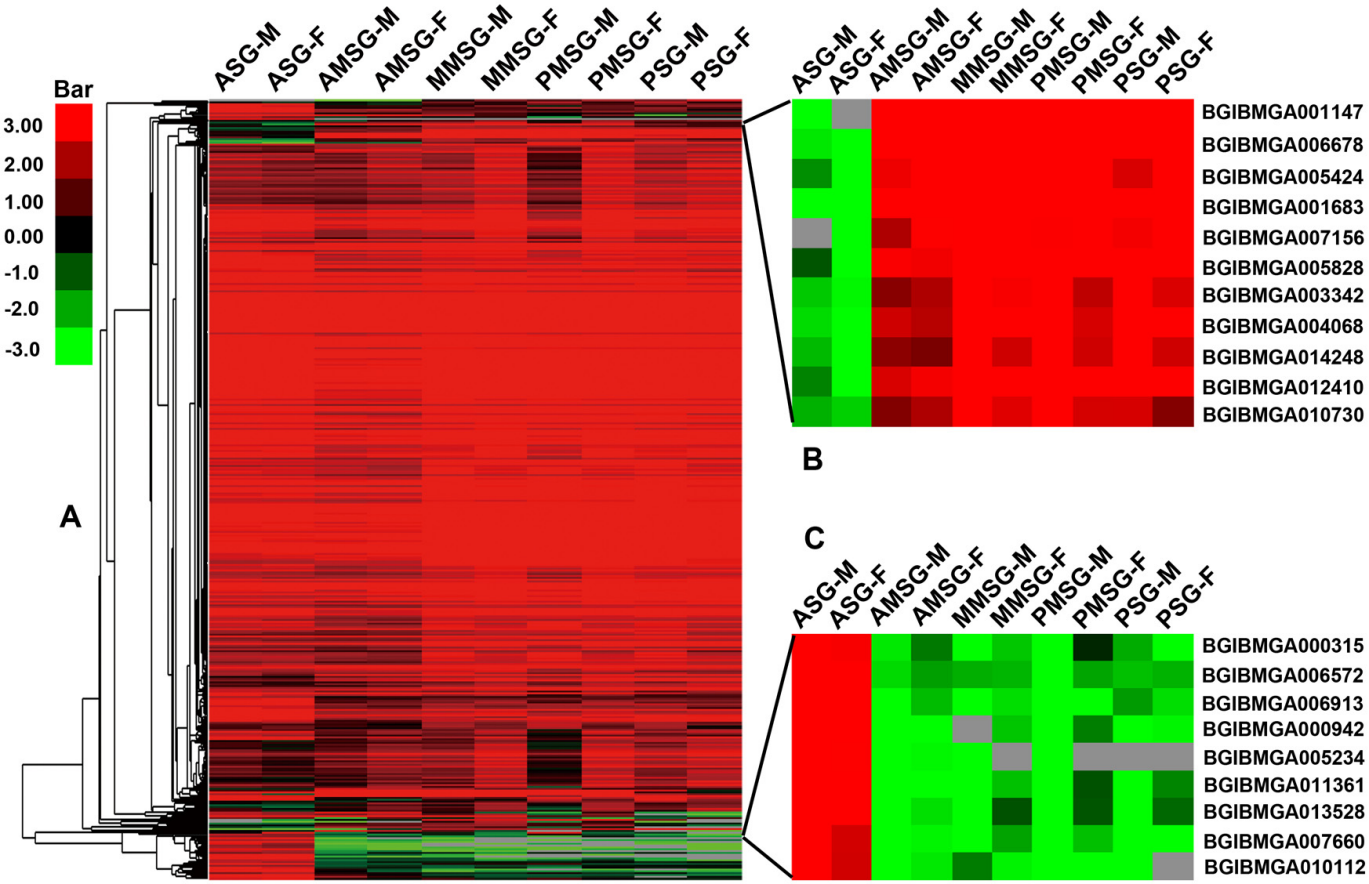
Hierarchical clustering of the expressed genes in different parts. (A) The expression profile of all expressed genes in the silk gland. (B) The expression profile of some genes with down-regulated expression in the ASG. (C) The expression profile of some genes with up-regulated expression in the ASG. The RNA-Seq data for clustering derived from the different parts of the silk gland based on RPKM values. The red bands indicate genes with high expression levels and the green bands indicate genes with low expression levels.

**Fig 2:**
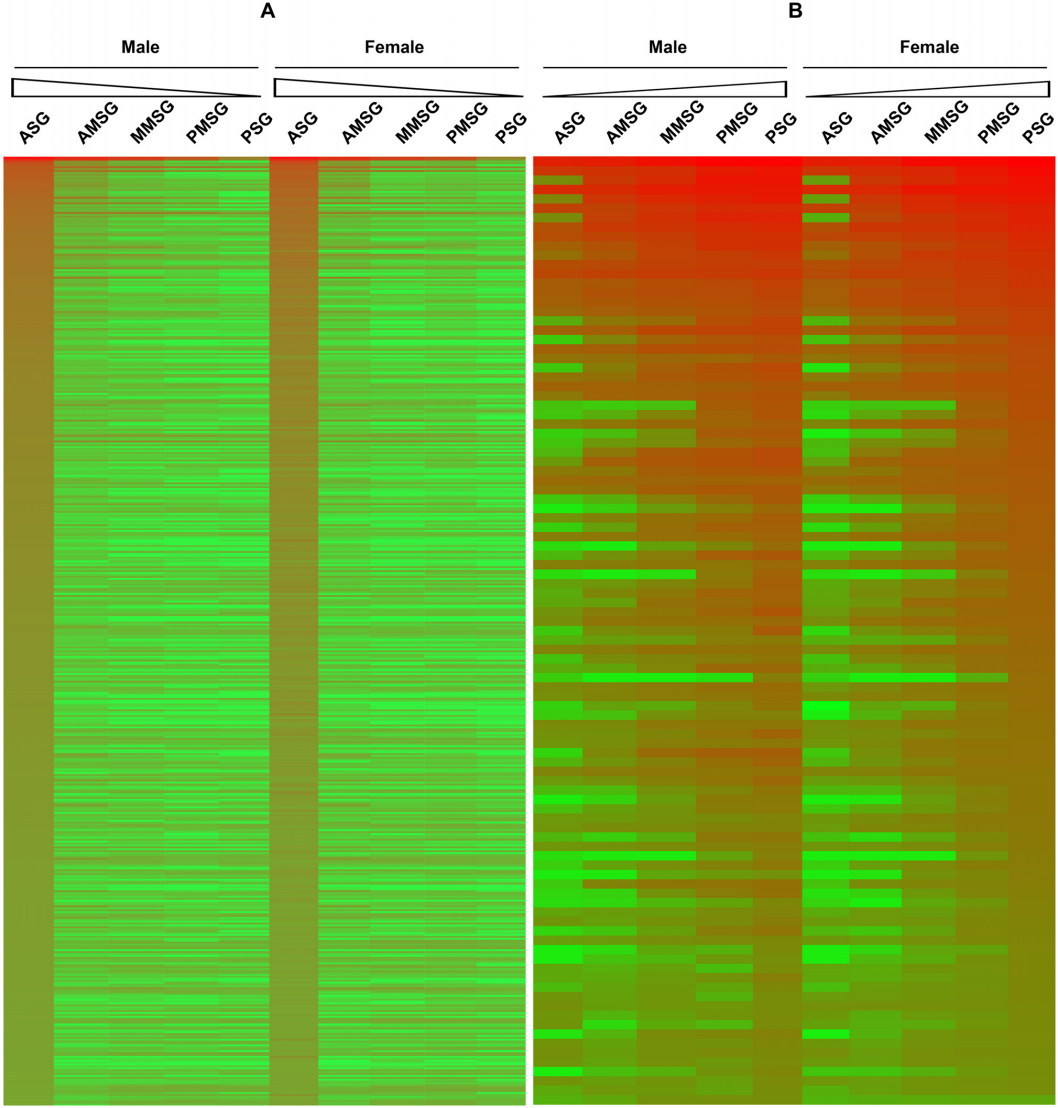
Gene expression gradient of the five silk gland sections. (A) Gene gradient of the 498 genes descending from the ASG to the PSG. (B) The 101 expressed genes crescent from the ASG to the PSG. The red bands indicate genes with high expression levels and the green bands indicate genes with low expression levels.

The expressed genes were screened and evaluated by mapping to the silkworm genome. RPKMs of all 14,623 silkworm genes (according to SilkDB) in ten different samples were calculated and analyzed (S2 Table). A total of 7419 genes were expressed in at least one sample (S3 Table). There were 5592 and 5339 genes expressed in the ASG of male and female silkworms, corresponding to 38.2% and 36.5% of all genes predicted in SilkDB, respectively [20]. Different numbers of genes were expressed among these five sections. Interestingly, the changes in the numbers of expressed genes in the males from all parts were similar to those in the females, reflecting the reliability of the RNA-Seq data (S4 Table). All the expressed genes were clustered using Cluster v3.0 (Fig 1) and the changes in expression level were similar to that in number of the expressed genes, again indicating that the RNA-Seq data is reliable. Silk proteins are synthesized in the PSG and MSG, then move into the ASG, and secreted finally. To unveil the gene expression regulation in the different sections, the gene expression gradient was determined from ASG to PSG (Fig 2, S5 Table). A total of 498 genes were expressed descending from the ASG to the PSG, and 101 genes were crescent from the ASG to the PSG. The dynamic gradients were similar in male and female larvae. In the descending gene gradient, 454 identified genes were expressed only in the ASG, including 35 cuticular genes, 29 transport-related genes, seven serpin-related genes, four chitin-related genes. These genes may play roles in packaging and secreting the silk proteins.

Twenty genes were selected to validate the RNA-Seq data, including five cuticle protein genes, two V-ATPase-related genes, three serpin-related genes, three glycolysis-related genes, three silk protein-related genes and four others (S6 Table). The gel electrophoresis results (S6 Table) indicated that the gene expression changes were similar to the RPKM changes, which provided strong verification for the RNA-Seq data.

### Up-regulated Genes in the ASG

**Fig 3:**
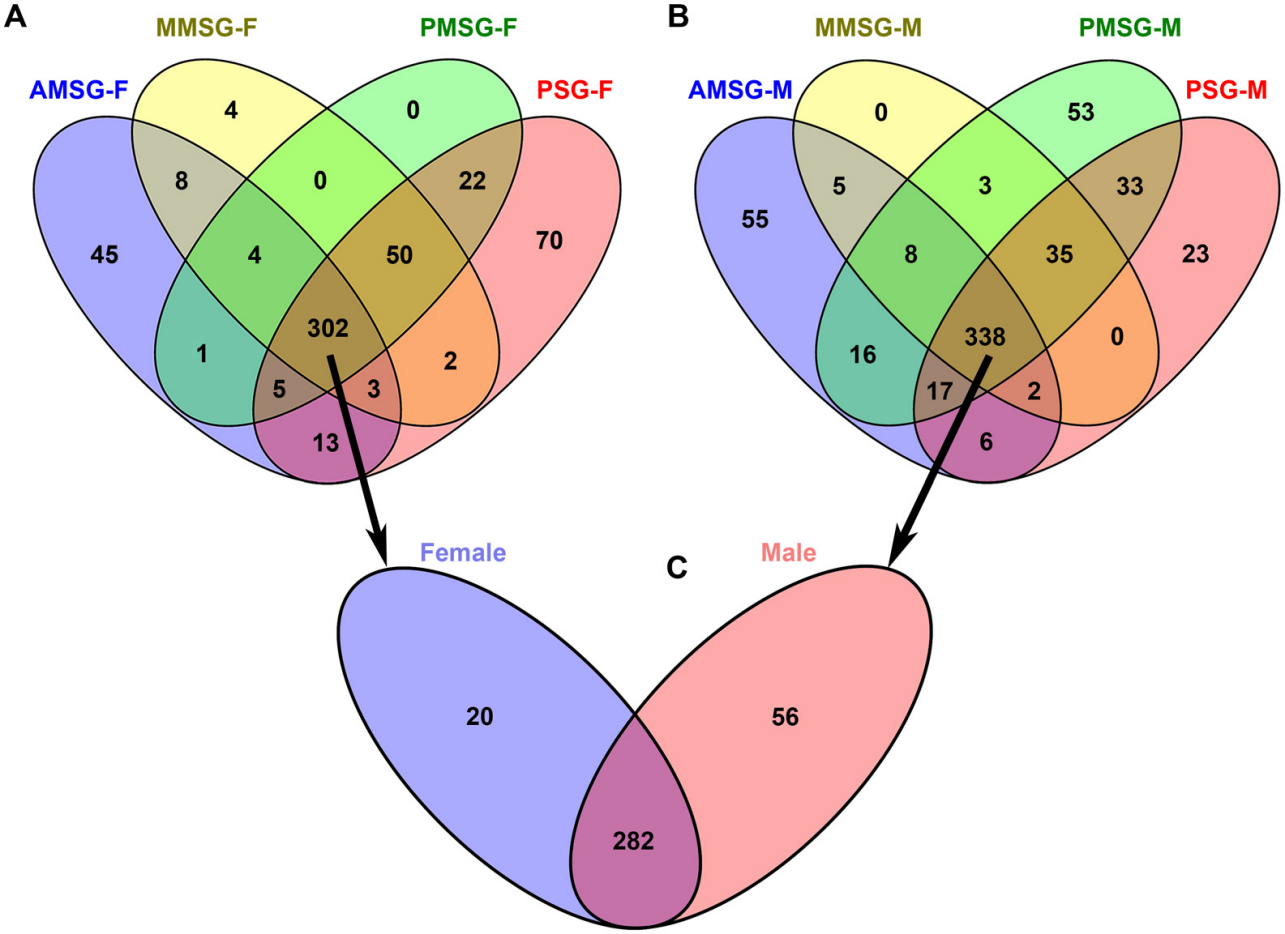
Venn diagram of the up-regulated genes between ASG and other parts of the silk gland. (A) Respective overlaps of up-regulated genes in the ASG vs other parts of the silk glands from female silkworms. (B) Respective overlaps of up-regulated genes in the ASG vs other parts of the silk glands from male silkworms. (C) Up-regulated genes in the ASG and other parts of silk glands from females and males.

**Fig 4:**
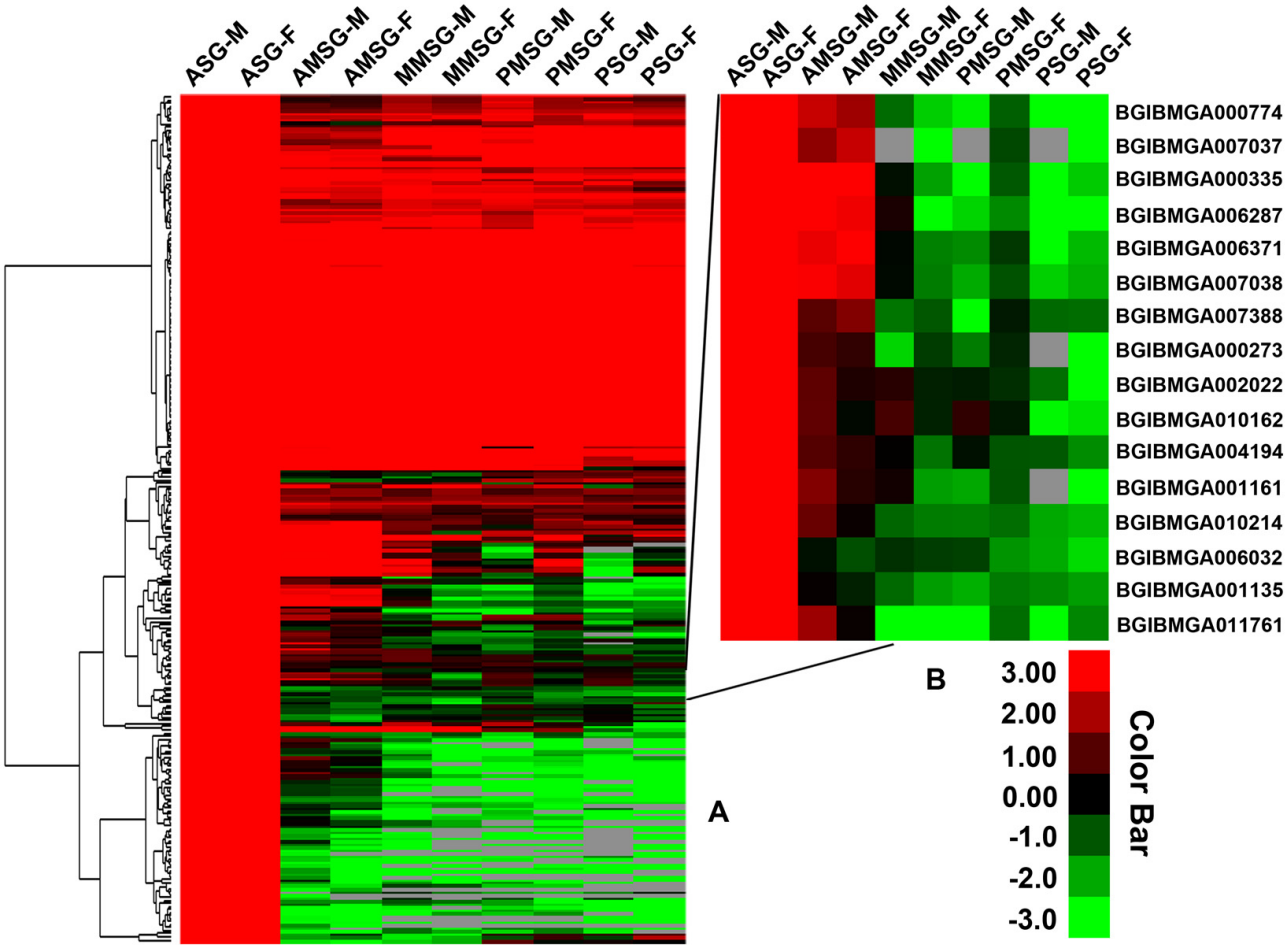
Hierarchical clustering of the up-regulated genes in the ASG vs other parts of the silk gland. (A) The 282 genes up-regulated in the ASG. (B) Example of several clusters, including 16 genes. The red bands indicate genes with high expression levels and the green bands indicate genes with low expression levels.

The genes were considered to be highly expressed when the change fold was ≥2 (P-value < 0.05 and FDR < 0.001). Compared with the other parts, the up-regulated genes in the ASG were screened out (Fig 3). In male silkworms, 447, 391, 503 and 454 genes were up-regulated in the ASG compared with AMSG, MMSG, PMSG and PSG, respectively. There were 381, 373, 384 and 467 genes up-regulated in females. There were 282 genes up-regulated in ASG compared with all the other parts (S7 Table). The 282 genes were clustered using Cluster v3.0 (Fig 4), and the changes in expression levels in males were similar to those in females.

### Annotations of the Up-regulated Expressed Genes

GO analysis was employed to annotate the 282 up-regulated genes, of which 143 (50.7%) were annotated. 147 GO accession pathways were enriched, of which 58 pathways were selected using the standard P-value < 0.01 and FDR < 0.001 (S8 Table). These pathways were categorized into three domains: biological process, cellular component and molecular function. In the biological process, there were 41 GO pathways: one glycolysis, one oxidative phosphorylation, two alcohol metabolism, five carbohydrate metabolism, 18 nucleoside or nucleotide metabolism, two ATP metabolism, nine ion-transport and three other pathways on energy metabolism. In the molecular function domain, one pathway was related to structural constituents of cuticle proteins, and the other 14 pathways concerned transport activity of ion, cation, hydrogen, proton or other substances. In the cellular component domain, two pathways were related to proton transporting. The relationships of these GO pathways were evaluated (S1, S2 and S3 Figs) and resulted in seven terminal pathways: ‘ATP Synthesis coupled proton transport’, ‘Glycolysis’, ‘Hydrogen ion transporting ATP synthase activity, rotation mechanism’, ‘Proton-transporting ATPase activity, rotation mechanism’, ‘Structural constituent of cuticle’, ‘Serine-type endopeptidase inhibitor activity’ and ‘Proton-transporting two-sector ATPase complex’. These terminal pathways focused on ‘Ion-transporting’, ‘Energy metabolism’, ‘Proteinase inhibitor’ and ‘Cuticular proteins’, which are closely related to the formation and spinning of silk fibers.

**Fig 5:**
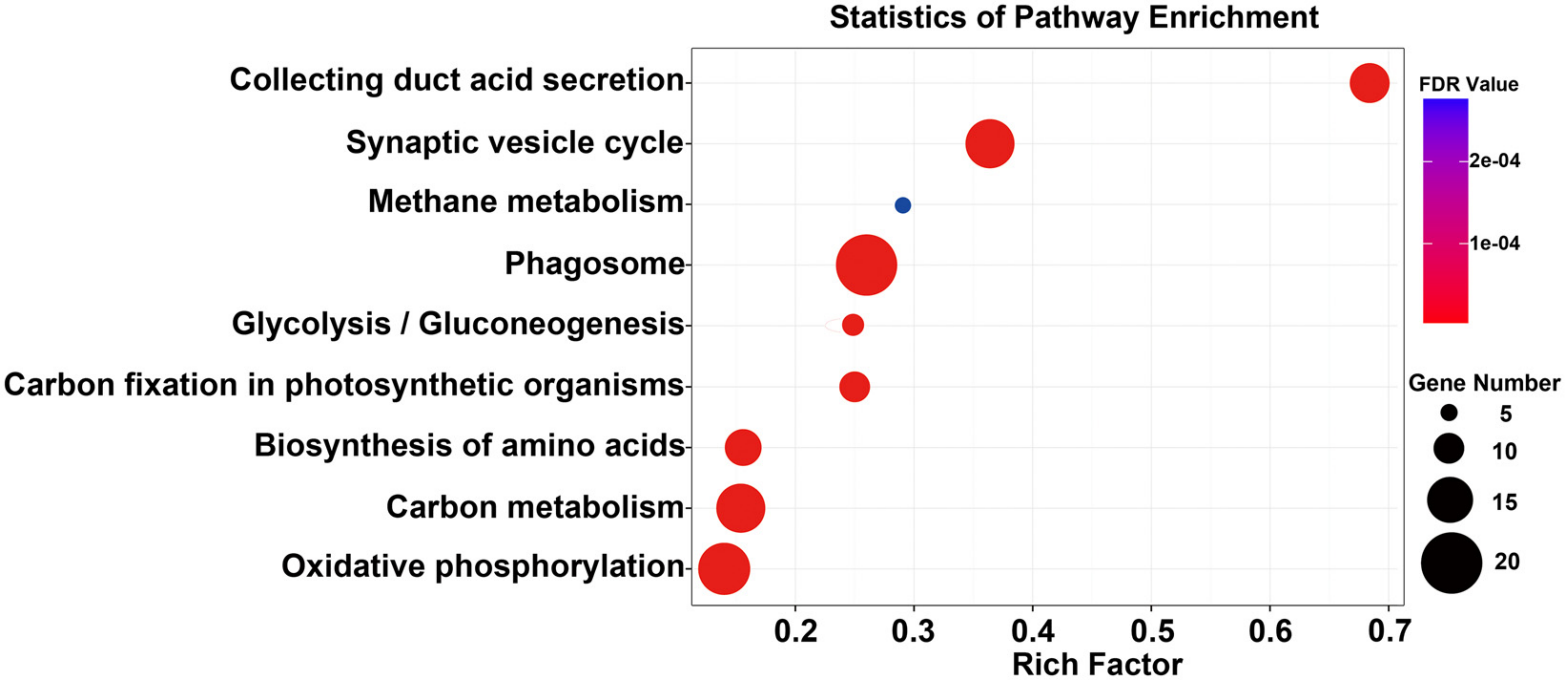
Scatter plot of enriched KEGG pathways for the 282 up-regulated genes in the ASG. The “rich factor” shows the ratio of the number of the up-regulated genes to the total gene number in certain pathways. All pathways had FDR ≤ 0.001.

KEGG was also used to annotate the up-regulated genes. A total of 96 pathways were enriched (S9 Table), of which nine pathways met the criteria of P-value < 0.05 and FDR < 0.001 (Fig 5), including six pathways for ‘Energy metabolism’, two for ‘pH controlling’ and one for ‘transport’, which was similar to the GO analysis results. The liquid silk proteins, which are synthesized in the PSG and MSG, move to the ASG, and are then spun out. In the ASG, the gel pattern silk proteins were transformed to soliquid—a better status for silk spinning. In this process, the pH gradient and cations play important roles [21, 22]. The pH decreases along the lumen of the PSG to the ASG, and affects the assembly of silk [23]. Lower pH and Ca^2+^ concentration, and higher potassium ion (K^+^) concentration, promote the formations of beta-sheets; however, higher pH and Ca^2+^, and lower K^+^, maintain the ‘random coil’ conformations of typical silk [24, 25]. A study of six metal elements—K, Ca, sodium (Na), magnesium (Mg), copper (Cu) and zinc (Zn)—at different stages in the silk secretory pathway showed that Mg^2+^, Cu^2+^ and Zn^2+^ induced the conformation transition of silk fibroin to beta-sheets, Ca^2+^ affected the formation of a stable protein network (gel) and Na^+^ and K^+^ broke down the protein network [4]. To obtain a perfect cocoon, the silkworm has to efficiently control pH and cations, and spin the silk proteins out smoothly. The enriched pathways related to transport and energy metabolism in the ASG that were detected in the present study provide an important reference for explaining the mechanism of the silkworm’s control of the internal environment to machine silk fibers.

### Genes Involved in the Ion-transporting Pathway

Ion transport was a main pathway identified in this study. This pathway is very important for silk formation [4, 9, 26]. After synthesis in the PSG, the fibroin moves to the MSG, and is covered by sericin, and then the liquid fibroin and sericin are transported into the ASG, where they are processed, from gel pattern to soliquid, which is better for spinning. In the present study, 18 genes were enriched in ion-transporting pathways (Table 2). SilkDB showed that 16 genes encoded different subunits of the V-type ATPase—responsible for ATP metabolism, usually in rotational catalysis mode, started by a proton gradient [27]. V-type ATPase was identified as a proton-translocating protein [28]. Of the 18 genes, BGIBMGA002241 and BGIBMGA008295 were reported by Wang [29], and V-ATPase may be involved in the generation and maintenance of the acidic environment in these two sections. The other 16 genes were found to be up-regulated in the ASG for the first time. It is exciting to find that so many genes were up-regulated in the ASG, which suggested that the transition of silk protein conformation could not occur without ion transport, and that more ion transport activity may occur in the ASG compared with other parts. Changing the ion concentration or adjusting the ATPase activity would be a useful method to achieve silk fiber with special features, such as higher intensity and flexibility. Additionally, the transferrin precursor was detected in the ion-transport pathway, suggesting that iron was involved in silk fiber formation and spinning, and perhaps aided the antibacterial activity of silk fibers, which is an important feature of the cocoon for protecting the pupae.

**Table 2:** Up-regulated genes that are involved in ion-transport pathwayin ASG.

GeneID	Gene	E-value	Identity
BGIBMGA000231-PA	vacuolar ATP synthase 21 kDa proteolipid subuni	4.00E-139	100
BGIBMGA000596-PA	vacuolar ATP synthase subunit H	0	98.48
BGIBMGA001302-PA	vacuolar H+ ATP synthase 16 kDa proteolipid subunit	2.00E-95	93.94
BGIBMGA002241-PA	vacuolar ATPase B subunit	0	100
BGIBMGA003196-PA	V-type proton ATPase subunit d	0	99.71
BGIBMGA008011-PA	vacuolar ATP synthase subunit F	2.00E-83	100
BGIBMGA008295-PA	vacuolar ATP synthase catalytic subunit A	0	93.52
BGIBMGA008542-PA	vacuolar ATP synthase subunit D	1.00E-160	100
BGIBMGA008879-PA	vacuolar ATPase subunit H	0	66.85
BGIBMGA010246-PA	vacuolar ATP synthase subunit e	5.00E-38	98.57
BGIBMGA010247-PA	vacuolar ATP synthase subunit E	8.00E-103	99.34
BGIBMGA013963-PA	vacuolar ATPase subunit C	3.00E-60	97.94
BGIBMGA013964-PA	vacuolar ATPase subunit C	0	95.44
BGIBMGA014403-PA	PREDICTED: V-type proton ATPase subunit S1-like	0	100
BGIBMGA014507-PA	PREDICTED: V-type proton ATPase subunit e-like	5.00E-24	97.96

### Genes that are Involved in Glycolysis

Glycolysis is one of the most important ways for organisms to produce ATP required for biological processes. Two ATP molecules are generated when one molecule of glucose is catalyzed into two molecules of pyruvate through glycolysis. Here, the glycolysis pathway was enriched from the up-regulated genes in the ASG (Table 3), which is similar to the results in earlier research using the shotgun proteomic method, in which many ATPases were identified in ASG cells [9]. Of the related genes, the glyceraldehyde-3-phosphate dehydrogenase (SilkDB No. BGIBMGA007490), mitochondrial aldehyde dehydrogenase (SilkDB No. BGIBMGA010403) and arginine kinase genes (SilkDB No. BGIBMGA005812) were discovered in both the present study and shotgun results, but the latter two genes were not enriched in the glycolysis pathway. That more glycolysis-related genes were found in the ASG suggests that glycolysis was more active in the ASG than in the MSG and PSG. Silk proteins are produced in the PSG and MSG, but processed and transformed in the ASG, where the liquid fibroin and sericin are transformed into nematic liquid crystalline phase and secreted out of the silkworm. Energy is necessary for such processes as ion-transport, pH regulation and mechanical treatment, which is perhaps the reason for glycolysis enrichment.

**Table 3:** The up-regulated genes that are associated with glycolysis in the ASG.

GeneID	Gene	E-value	Identity
BGIBMGA002508-PA	PREDICTED: pyruvate kinase-like isoform X1	0	99.33
BGIBMGA004221-PA	glucose-6-phosphate isomerase	0	99.81
BGIBMGA005493-PA	putative enolase protein	0	99.74
BGIBMGA007490-PA	glyceraldehyde-3-phosphate dehydrogenase	0	100
BGIBMGA007681-PA	PREDICTED: phosphoglycerate kinase-like	0	100
BGIBMGA013021-PA	fructose 1,6-bisphosphate aldolase	0	100

### Genes Involved in the Serine-type Endopeptidase Inhibitor Activity

Serine-type endopeptidase inhibitor activity was another enriched pathway—with six up-regulated serine protease inhibitors in the ASG (Table 4), similar to the shotgun proteomics results [9]. The serpin family members 2 and 5 (SilkDB Nos. BGIBMGA007720 and BGIBMGA013849) were also detected in the shotgun study, but the members 10, 11, 12 and 32 were only found in the present study. These protease inhibitors could protect silk proteins from being degraded before secretion [30]. Two novel serine protease inhibitors were detected in the MSG and PSG, and transported to the ASG 8. Tyrosine inhibitors were found to be capable of preventing L-fibroin chains from being degraded [31]. Another study reported that more serine protease inhibitor genes were expressed in silk glands than in nine other tissues, and revealed that serine protease inhibitors in the silk gland belonged to the silkworm-specific families [23]. In the present study, many serine protease inhibitors were up-regulated in the ASG compared to other parts, which are likely a long-term evolutionary result in silkworm, and these inhibitors would function as monitors/evaluators restraining the activity of serine protease during the process of formation and spinning of silk fibers. A balance between the inhibitors and the protease would be a helpful strategy to maintain the silk quality.

**Table 4:** Genes associated with endopeptidase inhibitor activity up-regulated in ASG.

GeneID	Gene	E-value	Identity
BGIBMGA007720-PA	serine protease inhibitor 2	0	99.1
BGIBMGA010213-PA	serine protease inhibitor 11 precursor	0	98.68
BGIBMGA010214-PA	serine protease inhibitor 10 precursor	0	99.79
BGIBMGA010216-PA	serine protease inhibitor 12	0	97.67
BGIBMGA013848-PA	serine protease inhibitor 32 precursor	0	100
BGIBMGA013849-PA	serine protease inhibitor 5 precursor	0	99.42

### Genes Associated with the Synthesis of Cuticle Proteins

Cuticular proteins are main components of the insect cuticle, which plays an important role in many biological processes, including protecting the insect’s body from invasion of pathogens, physical injury and insecticide penetration [32]. Apart from the epidermal tissues, the cuticular proteins are also expressed in many intracorporal organs. In the present study, 53 genes encoding cuticular proteins were found in the ASG (S10 Table), 28 of which were up-regulated in the ASG, and 16 were enriched in the ‘Structural constituent of cuticle’ pathway (Table 5). Based on whether or not the conserved R&R (Rebers and Riddiford) consensus and other motifs were presented, cuticular proteins were categorized into several groups: including RR-1 (proteins from soft cuticles), RR-2 (proteins from hard cuticles) [33, 34], RR-3 (with the 75-residue motif) [35], CPF and CPFL [36], Tweedle [37] and others [32, 38]. Of the 53 genes, 12 were RR-1 protein, 18 were RR-2 protein, two were RR-3 protein, four were GRP, two were Tweedle and 15 were CPH genes. Interestingly, seven of the 53 genes were not found in a previous genome-wide annotation analysis [39].

**Table 5:** Up-regulated genes that are involved in cuticle protein synthesis in the ASG.

GeneID	Gene	E-value	Identity
BGIBMGA000273-PA	cuticular protein RR-2 motif 91 precursor	2E-75	100
BGIBMGA000277-PA	cuticular protein RR-2 motif 87 precursor	2E-105	100
BGIBMGA000278-PA	cuticular protein RR-2 motif 86 precursor	3E-106	100
BGIBMGA000281-PA	TPA: putative cuticle protein	5E-126	100
BGIBMGA000334-PA	cuticular protein RR-1 motif 37 precursor	7E-91	100
BGIBMGA000335-PA	putative cuticle protein	0	100
BGIBMGA000336-PA	cuticular protein RR-1 motif 34 precursor	4E-146	100
BGIBMGA010142-PA	TPA: putative cuticle protein	6E-123	100
BGIBMGA010143-PA	cuticular protein RR-2 motif 70 precursor	4E-124	100
BGIBMGA010145-PA	cuticular protein RR-2 motif 67 precursor	2E-118	100
BGIBMGA010231-PA	cuticular protein RR-2 motif 68 precursor	0	100
BGIBMGA010232-PA	cuticular protein RR-2 motif 69 precursor	4E-100	100
BGIBMGA010320-PA	cuticular protein RR-2 motif 140 precursor	0	100
BGIBMGA010483-PA	cuticular protein RR-1 motif 54 precursor	0	98.7
BGIBMGA010500-PA	cuticular protein hypothetical 28 precursor	0	100
BGIBMGA013163-PA	cuticular protein RR-1 motif 56 precursor	9E-129	100

The previous shotgun proteomics analysis found only 32 cuticular genes expressing in the ASG, much less than found in the present study [9], which confirmed the advantage of the RNA-Seq technology over the proteomics technique. Additionally, two genes by the shotgun method did not encode cuticular proteins according to the SilkDB, and this should be verified further.

The ASG is the region where the liquid silk proteins are transformed to solid thread, and the silk fibers are spun out steadily. All these processes require a strong and flexible lumen wall, which is made of chitin and cuticle proteins. Chitin was found to exist in the silk gland ducts of both the spider and the silkworm [40]; and the property of cuticular proteins and the mechanism by which they interact with chitin have been extensively studied [33, 41, 42]. The extended R&R consensus was declared a chitin-binding domain, in which aromatic residues may play a role in binding chitin [43].

Chitin may play a role in providing a lubricant in lumen for silk spinning, and the chitin component in the spinneret should be surveyed in the future. Additionally, the forceful transport of energy, ions and protons in the ASG during silk transformation and spinning requires a membrane with good permeability. The cuticular proteins and chitin may provide the ASG with this feature.

### Sexual Differences in Gene Expression in the Silk Gland

**Fig 6:**
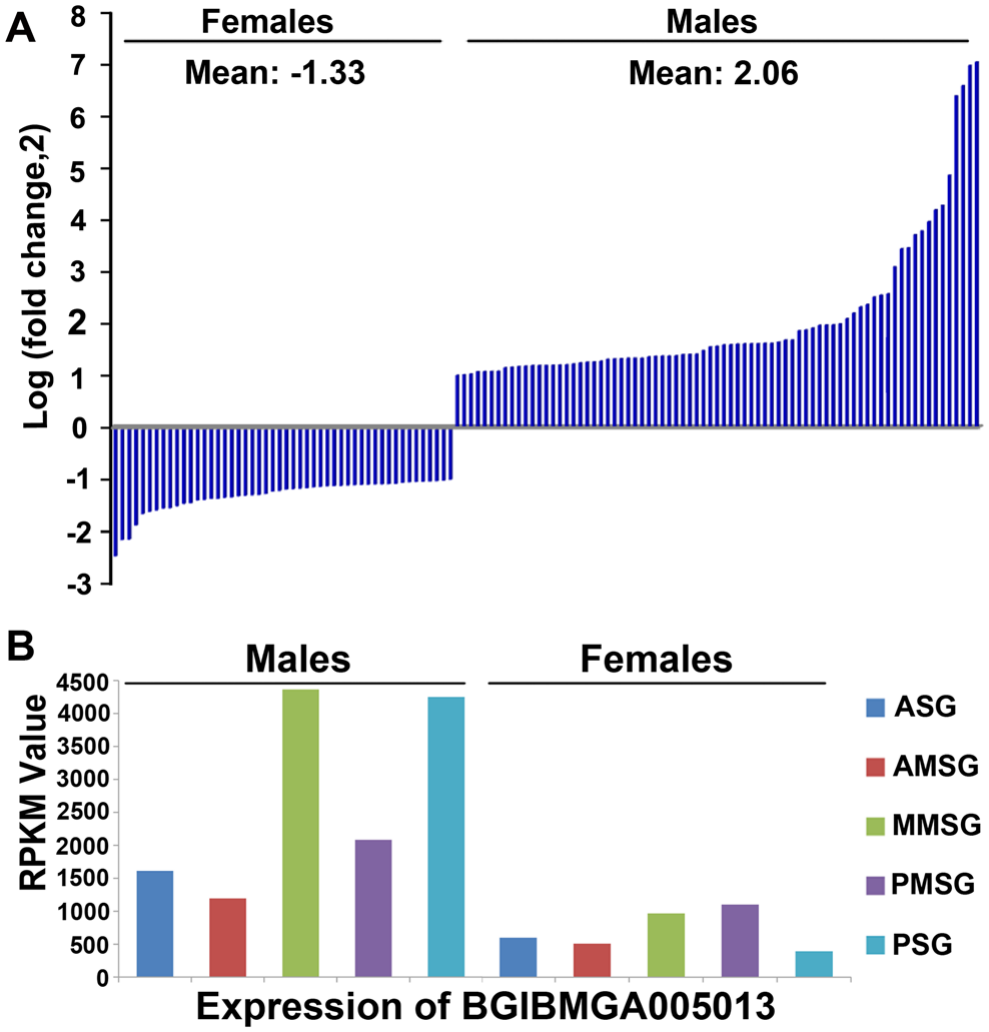
Expression patterns of up-regulated genes in males and females. (A) PSG as an example of the gene expression pattern based on the fold-change between males and females. There are more up-regulated genes in the PSG of males than of females. (B) Expression pattern of the gene BGIBMGA005013.

To explore the sexual differences of silk glands at the transcriptomic level, we surveyed the DEGs of different parts of silk glands between the male and female silkworms. A total of 210 genes were expressed differently between males and females under the criteria fold-change ≥ 2, P-value < 0.05 and FDR < 0.001 (S11 Table). Nineteen genes were expressed differently in ASG, out of which 15 genes were up-regulated in males, and the other four genes were up-regulated in females. In MMSG and PSG, there were 105 and 127 DEGs, respectively, much more than in ASG (S11 Table), suggesting that the difference during the silk protein synthesis in MSG and PSG may play more important roles in the sexual cocoon variation [44] than in the processing in ASG. The number and type of DEGs were distinct between the genders in MMSG and PSG. In MMSG, more DEGs were found in the female (Male/Female: 29/76), but it was 77/50 in PSG (Fig 6). In females, 27 genes were up-regulated in both the PSG and MMSG. In males, three genes were up-regulated in both the ASG and MMSG, and 11 genes were up-regulated in both the MMSG and PSG. Interestingly, only one gene (BGIBMGA005013) was up-regulated in all regions of the males (S11 Table). In PSG, three genes were reported in the former study based on the microarray data [10], including the peptide transporter related gene (BGIBMGA000496), the mitochondrial import inner membrane translocase encoding gene (BGIBMGA002215) and the heat shock protein encoding gene (BGIBMGA004612). Peptide transporters can mediate the trans-membrane transportation of the peptides [45], and the mitochondrial import inner membrane translocase participates in transporting the trans-membrane proteins into the mitochondrial inner membrane [46], which perhaps provide some clues for further study on the molecular mechanism of the sexual differences of the silk fibers.

## Conclusion

We explored the gene expression profiles in the silkworm ASG, AMSG, MMSG, PMSG and PSG. A total of 7419 genes were identified, out of which 5592 and 5339 were expressed in ASG from male and female silkworms, respectively. Compared with other parts, 282 genes, including 28 cuticle protein encoding genes, were up-regulated in the ASG. Annotation of these genes suggested that ion transportation was a vital pathway in the transformation of silk protein structure, and the cuticle proteins and serpins encoding genes played important roles in the formation and spinning process of the silk fibers, and all of these are energy-consuming processes.

## Supporting Information

S1 Fig Pathways involved in the domain biological process. Colors indicate the significance of the pathways. Arrows indicate the relationships through the pathways.
(TIF)

S2 Fig Pathways involved in the domain molecular function. Colors indicate the significance of the pathways. Arrows indicate the relationships through the pathways.
(TIF)

S3 Fig Pathways involved in the domain cellular component. Colors indicate the significance of the pathways. Arrows indicate the relationships through the pathways.
(TIF)

S1 Table Primers of the genes for RT-PCR validation. (XLSX)

S2 Table RPKMs of 14,623 silkworm genes in different parts of the silk gland. (XLS)

S3 Table The expressed genes in the silk gland. Each gene had RPKM ≥ 5 in at least one tissue.
(XLS)

S4 Table Numbers of expressed genes in different parts. (XLSX)

S5 Table Genes with expression gradient from ASG to PSG. (XLSX)

S6 Table Results of RT-PCR validation. (XLSX)

S7 Table Genes up-regulated in the ASG. (XLSX)

S8 Table GO pathways of the up-regulated genes in the ASG. (XLSX)

S9 Table KEGG pathways of the up-regulated genes in the ASG. (XLSX)

S10 Table The genes encoding cuticular proteins expressed in the ASG. ‘Y’ indicates the gene was found in the related report, and ‘N’ indicates not found.
(XLS)

S11 Table Gene expression difference between the male and the female. (XLSX)
