# Supplementary figures and images for: Transcriptomic Analysis of the Anterior Silk Gland in the Domestic Silkworm (Bombyx mori) – Insight into the Mechanism of Silk Formation and Spinning
Source: PLoS One. 2015 Sep 29;10(9):e0139424. doi: 10.1371/journal.pone.0139424 (PMC4587926; doi:10.1371/journal.pone.0139424)

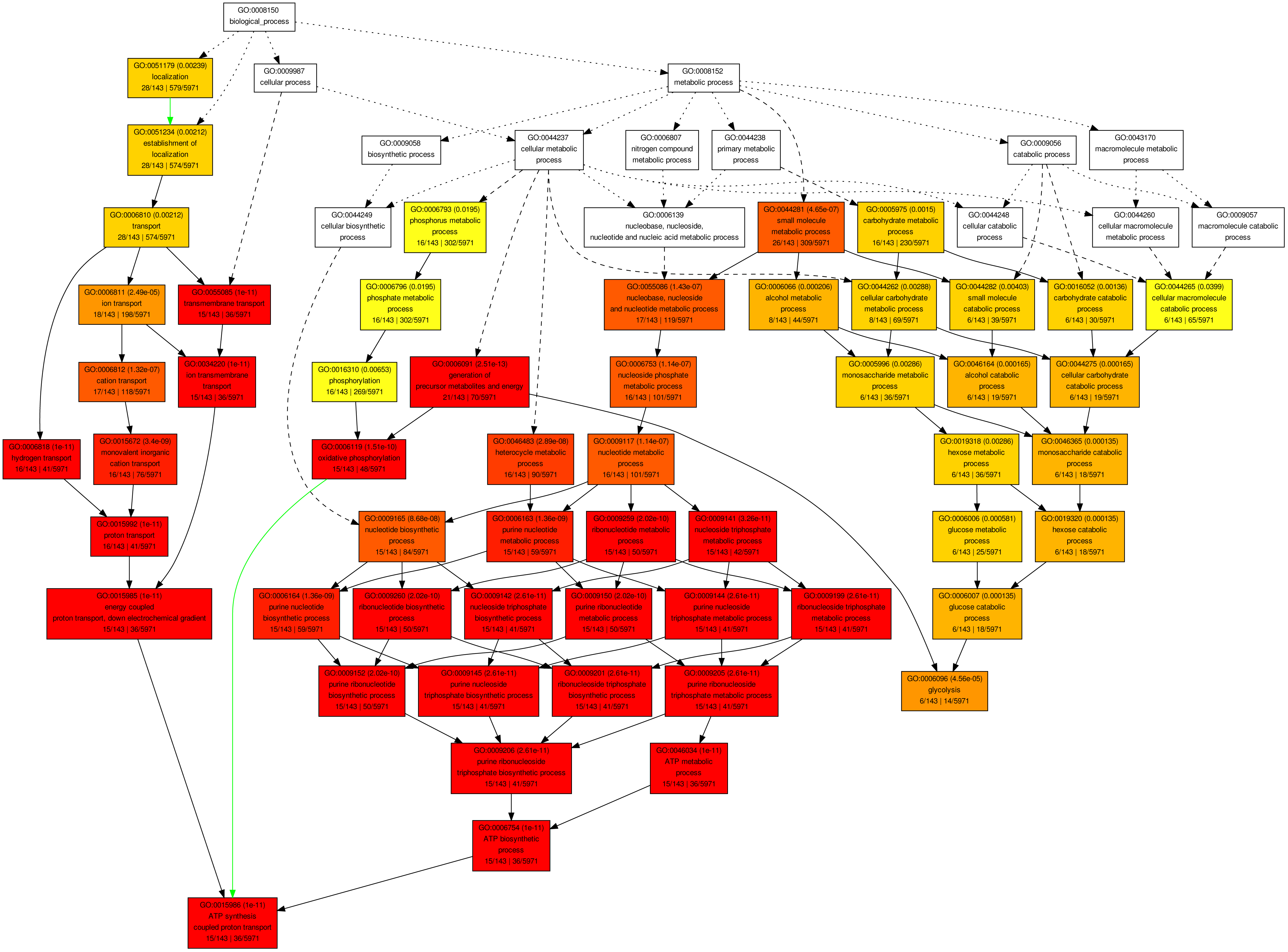

Supplement: S1 Fig — Colors indicate the significance of the pathways. Arrows indicate the relationships through the pathways. (TIF) [file pone.0139424.s001.tif]

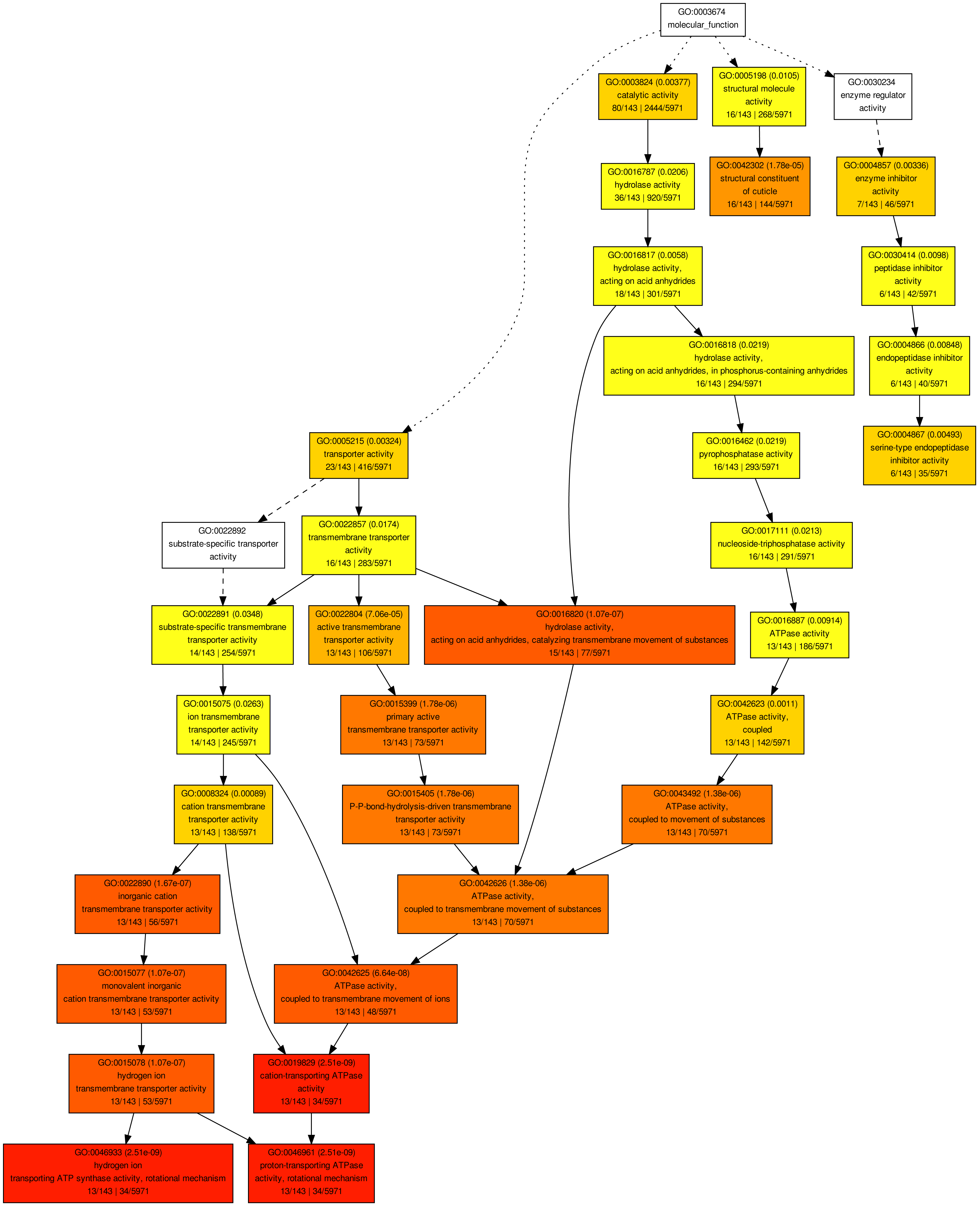

Supplement: S2 Fig — Colors indicate the significance of the pathways. Arrows indicate the relationships through the pathways. (TIF) [file pone.0139424.s002.tif]

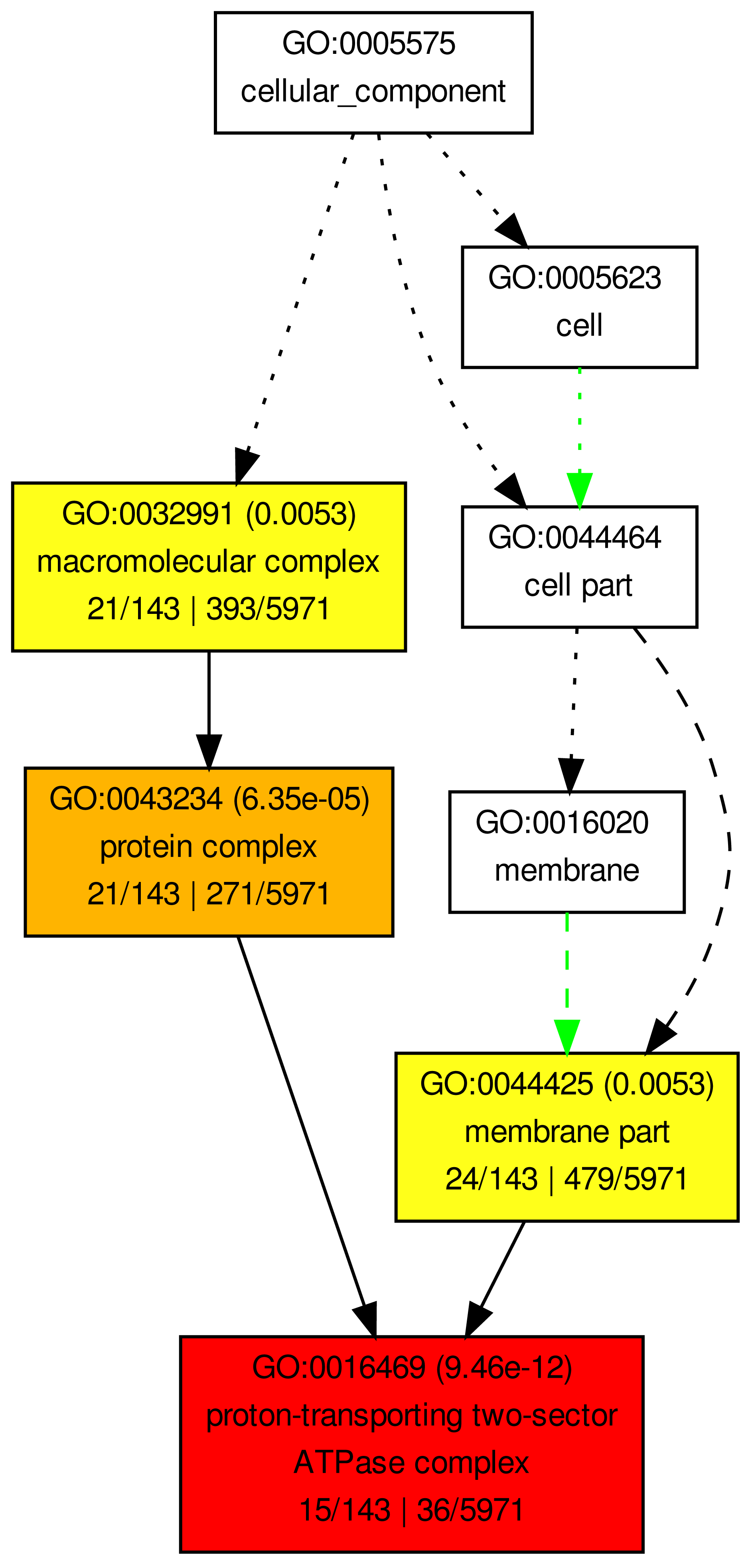

Supplement: S3 Fig — Colors indicate the significance of the pathways. Arrows indicate the relationships through the pathways. (TIF) [file pone.0139424.s003.tif]
